# Supplementary material for: AutoSpill is a principled framework that simplifies the analysis of multichromatic flow cytometry data
Source: Nat Commun. 2021 May 17;12:2890. doi: 10.1038/s41467-021-23126-8 (PMC8129071; doi:10.1038/s41467-021-23126-8)

Supplementary Technical Note

AutoSpill - Basic algorithm

The basic algorithm calculates a first approximation to the spillover matrix, and then it refines it iteratively.

Input: Collection of c single-color controls $\{\mathbf{X}_i\}$, $i = 1, \ldots, c$, each one being a matrix with n_i rows (events) and c columns (channels or dyes).

Output: Spillover matrix $\mathbf{S}$, a square matrix with c rows and columns, and the collection of compensated controls $\{\mathbf{Y}_i\}$, $i = 1 \ldots c$.

Parameters: Upper bound in the error of slopes required to achieve convergence, ε.

Algorithm:

1. For each single-color control $\mathbf{X}_i$,

    For each channel $j \neq i$,

        calculate robust linear model $\mathbf{X}_i[\star, j] \sim \mathbf{X}_i[\star, i]$ and obtain slope s_{ij}.

2. Build initial spillover matrix $\mathbf{S}^{(0)}$ as

$$\mathbf{S}^{(0)}[i,j] = \begin{cases} 1 & \text{if } i = j, \\ s_{ij} & \text{if } i \neq j. \end{cases}$$

3. Calculate initial compensation matrix $\mathbf{C}^{(0)} = (\mathbf{S}^{(0)})^{-1}$.

4. Obtain initial compensated controls $\{\mathbf{Y}_i^{(0)}\}$ by applying $\mathbf{S}^{(0)}$ on the controls $\{\mathbf{X}_i\}$.

5. Refine spillover matrix $\mathbf{S}^{(t)}$ and compensated controls $\{\mathbf{Y}_i^{(t)}\}$,
obtaining spillover matrix $\mathbf{S}^{(t+1)}$ and compensated controls $\{\mathbf{Y}_i^{(t+1)}\}$,
until convergence

    5.1. For each compensated single-color control $\mathbf{Y}_i^{(t)}$,

        For each channel $j \neq i$,

            calculate robust linear model $\mathbf{Y}_i^{(t)}[\star, j] \sim \mathbf{Y}_i^{(t)}[\star, i]$ and obtain slope $e_{ij}^{(t)}$.

    5.2. Build matrix of slope errors $\mathbf{E}^{(t)}$ as

$$\mathbf{E}^{(t)}[i,j] = \begin{cases} 0 & \text{if } i = j, \\ e_{ij}^{(t)} & \text{if } i \neq j. \end{cases}$$

    5.3. Calculate non-normalized spillover matrix $\mathbf{U}^{(t+1)} = \mathbf{S}^{(t)} + \mathbf{E}^{(t)}\mathbf{S}^{(t)}$.

    5.4. Calculate normalized spillover matrix $\mathbf{S}^{(t+1)}$ by rows, as

        $\mathbf{S}^{(t+1)}[i, \star] = \mathbf{U}^{(t+1)}[i, \star] \,/\, \mathbf{U}^{(t+1)}[i, i]$.

    5.5. Calculate compensation matrix $\mathbf{C}^{(t+1)} = (\mathbf{S}^{(t+1)})^{-1}$.

    5.6. Obtain compensated controls $\{\mathbf{Y}_i^{(t+1)}\}$ by applying $\mathbf{C}^{(t+1)}$ on the controls $\{\mathbf{X}_i\}$.

    5.7. Convergence is attained when $\|\mathbf{E}^{(t)}\| < \varepsilon$.

6. At convergence, t^* being the last iteration, obtain

$\mathbf{S} = \mathbf{S}^{(t^*)}$,

$\mathbf{Y}_i = \mathbf{Y}_i^{(t^*)}$, $i = 1 \ldots c$.

AutoSpill – Implementation details

The matrix inversions in steps 3 and 5.5 are calculated with the `R` base function `solve()`. Note that the matrices $\mathbf{S}^{(t)}$ are close to the identity matrix, and therefore very well-conditioned, so numerical inversion is ensured to work well.

Compensation in steps 4 and 5.6 is carried out with the function `compensate()` of the `R` package `flowCore`.

To check convergence in step 5.7, the norm used to assess the size of the slope errors is $||\mathbf{E}^{(t)}|| = \max(|\mathbf{E}^{(t)}[i, j]|)$, and the maximum tolerable error at convergence is $\varepsilon = 10^{-4}$.

AutoSpill – Further refinements of the algorithm

The basic algorithm works very well with good, clean single-color controls, for which the error $||\mathbf{E}^{(t)}||$ decreases monotonously with t until convergence. For more problematic cases, for example controls with low number of cells or low signal in the main channel, several improvements have been introduced, which mostly increase the control over the convergence of the algorithm.

The calculation of the linear models (steps 1 and 5.1) is performed on untransformed data only at the beginning. When $||\mathbf{E}^{(t)}|| < \varepsilon' = 10^{-2}$, the calculation (already in step 5) switches to work in data transformed with the bi-exponential transformation. This accelerates convergence and helps with obtaining good compensation in the transformed space where it will be evaluated. The slope calculated in this transformed space is translated back into linear space, by using two extreme points in the regression line. This untransformed slope is in fact an underestimation of the correct slope in linear space, which ensures the stability of the algorithm.

There are cases with noisy controls, for which the algorithm reaches relatively good convergence with $||\mathbf{E}^{(t)}|| < 10^{-2}$, but not so far as $||\mathbf{E}^{(t)}|| < 10^{-4}$. For those cases, the algorithm stops by observing oscillations in $||\mathbf{E}^{(t)}||$, without signaling a no-convergence error. Given the monotonous behavior of $||\mathbf{E}^{(t)}||$, oscillations are easily detected when $\Sigma^{9}_{\tau=0} ||\mathbf{E}^{(t-\tau)}|| > 0$, by implementing a circular buffer storing the last 10 values of $||\mathbf{E}^{(t)}||$. Further, when oscillations are first detected, a smaller step size is tried in 5.3. By writing the re_nement of the spillover matrix as $\mathbf{U}^{(t+1)} = \mathbf{S}^{(t)} + \lambda\mathbf{E}^{(t)}\mathbf{S}^{(t)}$, λ is reduced from $\lambda = 1$ to $\lambda = 0.1$.

Source data. Source data required to generate the main display items.

Supplementary Figure 1: Representative example of progressive error reduction with AutoSpill compensation methodology. Mouse splenocytes were stained for intracellular cytokine production following mitogen stimulation using a BD FACSymphony with 28 fluorophores. Compensation was performed using single colour controls of the same specificity as in the full panel; that is to say, anti-cytokine antibodies were used for single color controls. (a) Data depicted prior to compensation. (b) Compensation was performed using the traditional compensation wizard in FlowJo 10.7.1, correcting only channel assignments. (c) Compensation performed using the FlowJo traditional compensation wizard, optimizing the output by manually correcting the gating and positive-negative selection. (d) Traditional compensation performed using AutoSpill gating. (e) Compensation using the matrix produced by the first iteration of the AutoSpill regression. (f) Compensation using the final convergence of the AutoSpill algorithm. Pseudo-colour represents cellular density.

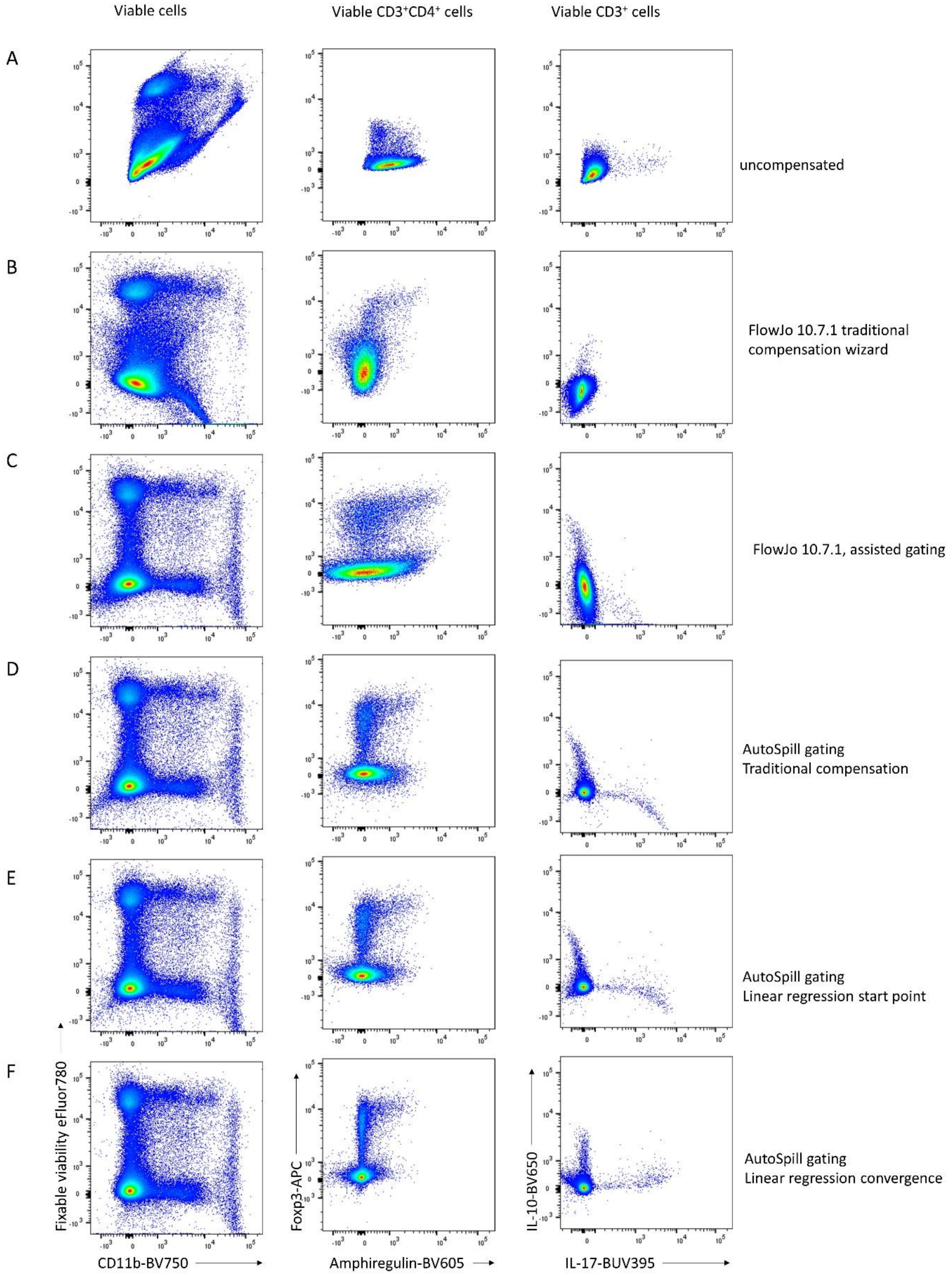

Viable cells
Viable CD3⁺CD4⁺ cells
Viable CD3⁺ cells
A
uncompensated
B
FlowJo 10.7.1 traditional compensation wizard
C
FlowJo 10.7.1, assisted gating
D
AutoSpill gating
Traditional compensation
E
AutoSpill gating
Linear regression start point
F
AutoSpill gating
Linear regression convergence
Fixable viability eFluor780
CD11b-BV750
Foxp3-APC
Amphiregulin-BV605
IL-10-BV650
IL-17-BUV395

Supplementary Figure 2: Changes in error calculation through the different tested methods across different numbers of single colour control cells. Single colour controls from dataset MM1 were randomly subsetted at 10 different cell numbers, with 10 subsamples with replacement taken for each cell number. (a) Spillover coefficients were calculated based on the sample subset, using AutoSpill gating and either: traditional (positive-negative) spillover calculation, the first stage of robust linear regression in AutoSpill, or the final convergence point of iterative robust linear regression (i.e., the full AutoSpill algorithm). (b) The number of iterative rounds used by AutoSpill to reach final convergence. Note, for cell subsets <150 cells, spillover coefficient calculations failed.

A

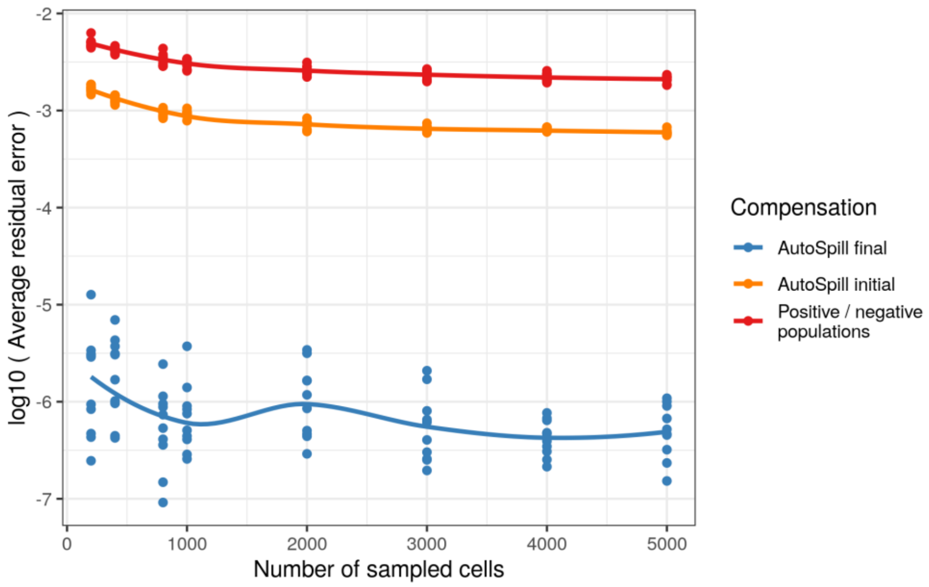

B

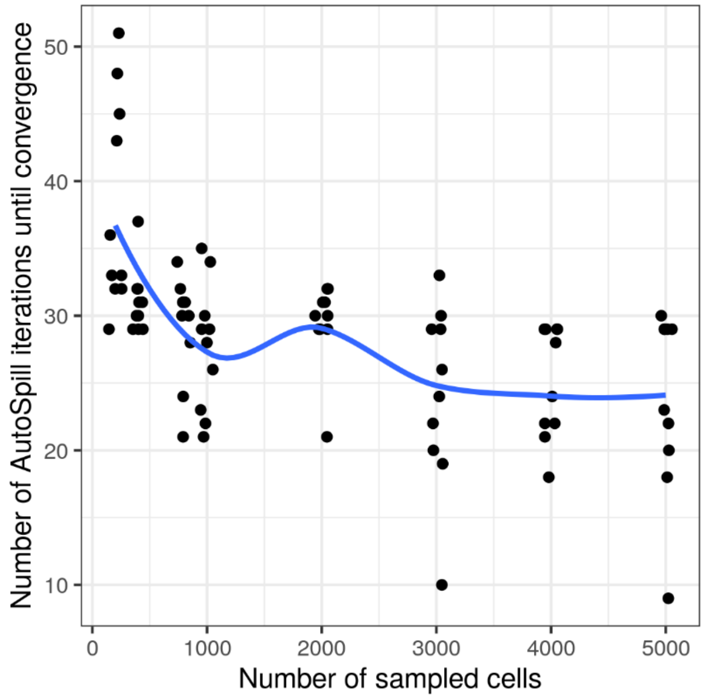

Supplementary Figure 3: Representative flow cytometry gating. Flow cytometric gating hierarchy. **(a)** Initial gating strategy for scatter and viability. **(b)** Gating hierarchy used for data shown in Figure 6D, brain and **(c)** spleen samples. Arrows represent subgating. (d) Gating hierarchy used for data shown in Figure 6E.

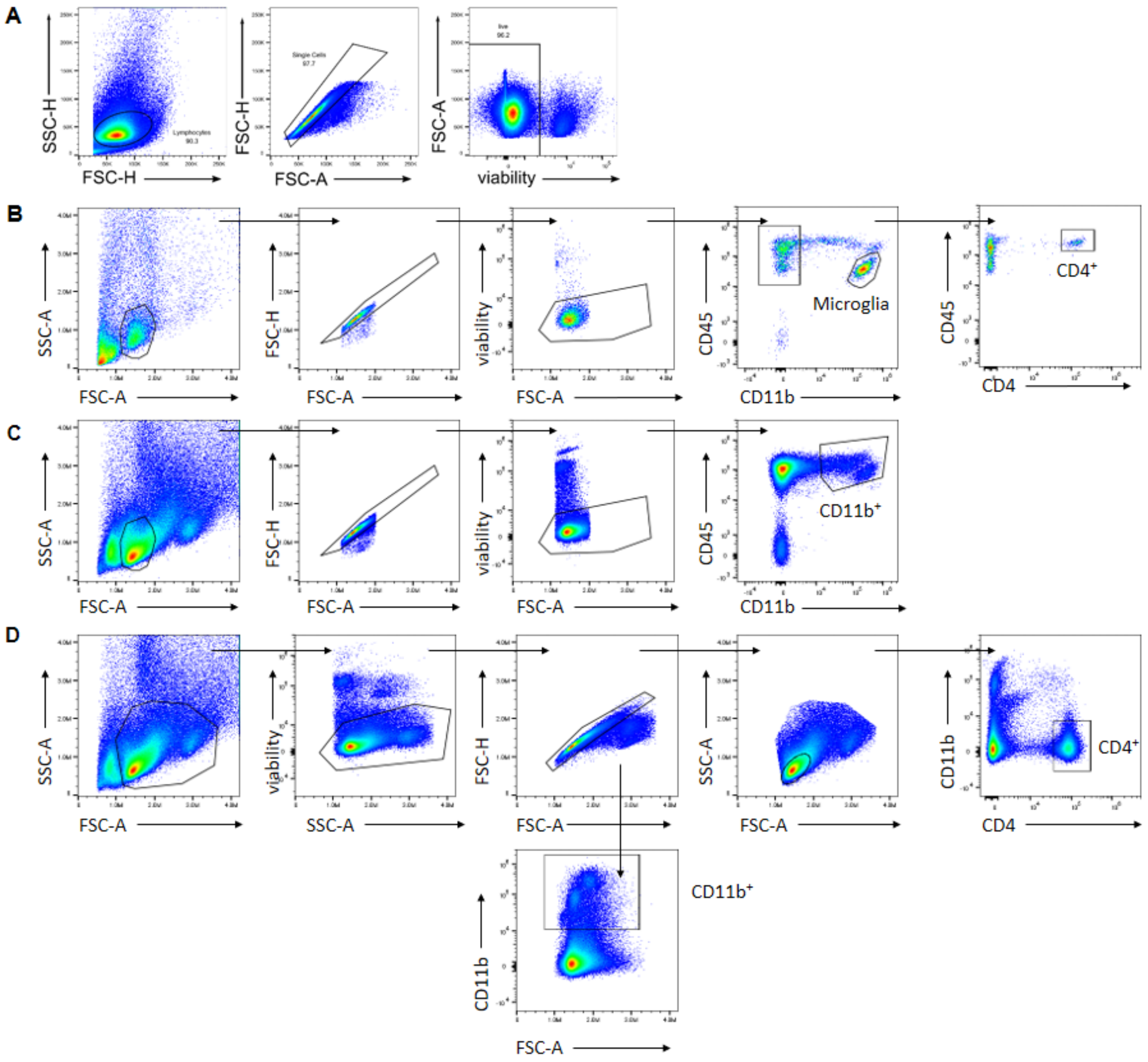

Supplement: Supplementary file 1 — Supplementary Information [file 41467_2021_23126_MOESM1_ESM.pdf]
