# Supplementary material for: AutoSpill is a principled framework that simplifies the analysis of multichromatic flow cytometry data
Source: Nat Commun. 2021 May 17;12:2890. doi: 10.1038/s41467-021-23126-8 (PMC8129071; doi:10.1038/s41467-021-23126-8)
Supplement: Supplementary file 2 — Description of Additional Supplementary Files [file 41467_2021_23126_MOESM2_ESM.pdf]

Description of Additional Supplementary Files

File Name: Supplementary Software 1
Description: Script for AutoSpill.
