# Supplementary material for: AutoSpill is a principled framework that simplifies the analysis of multichromatic flow cytometry data
Source: Nat Commun. 2021 May 17;12:2890. doi: 10.1038/s41467-021-23126-8 (PMC8129071; doi:10.1038/s41467-021-23126-8)
Supplement: Supplementary file 4 — Reporting Summary [file 41467_2021_23126_MOESM4_ESM.pdf]

nature research

Corresponding author(s): Adrian Liston

Last updated by author(s): Apr 14, 2021

Reporting Summary

Nature Research wishes to improve the reproducibility of the work that we publish. This form provides structure for consistency and transparency in reporting. For further information on Nature Research policies, see our Editorial Policies and the Editorial Policy Checklist.

Statistics

For all statistical analyses, confirm that the following items are present in the figure legend, table legend, main text, or Methods section.

n/a	Confirmed	
☐	☒	The exact sample size (*n*) for each experimental group/condition, given as a discrete number and unit of measurement
☐	☒	A statement on whether measurements were taken from distinct samples or whether the same sample was measured repeatedly
☒	☐	The statistical test(s) used AND whether they are one- or two-sided<br>*Only common tests should be described solely by name; describe more complex techniques in the Methods section.*
☐	☒	A description of all covariates tested
☐	☒	A description of any assumptions or corrections, such as tests of normality and adjustment for multiple comparisons
☒	☐	A full description of the statistical parameters including central tendency (e.g. means) or other basic estimates (e.g. regression coefficient) AND variation (e.g. standard deviation) or associated estimates of uncertainty (e.g. confidence intervals)
☒	☐	For null hypothesis testing, the test statistic (e.g. *F*, *t*, *r*) with confidence intervals, effect sizes, degrees of freedom and *P* value noted<br>*Give P values as exact values whenever suitable.*
☒	☐	For Bayesian analysis, information on the choice of priors and Markov chain Monte Carlo settings
☐	☒	For hierarchical and complex designs, identification of the appropriate level for tests and full reporting of outcomes
☒	☐	Estimates of effect sizes (e.g. Cohen's *d*, Pearson's *r*), indicating how they were calculated

Our web collection on statistics for biologists contains articles on many of the points above.

Software and code

Policy information about availability of computer code

Data collection	No software was used to collect the data.
Data analysis	AutoSpill was implemented in R v.3.6.3, using the packages flowCore v.1.52.1, flow-Workspace v.3.34.1, ggplot2 v.3.3.2, moments v.0.14, and RColorBrewer v.1.1-2. Tessellations were carried out with package deldir v.0.1-28, density estimations with packages MASS v.7.3-51.6, surface interpolations with package fields v.10.3, and spatial operations with packages sp v.1.4-2 and tripack v.1.3-9. Further details on packages specific to particular steps of the algorithm are listed in additional detail in the methods. Robust linear models were implemented with the package MASS v.7.3-51.6, with default parameters. FlowJo v10.6 and v10.7 were used, as noted in Figure legends. Source code for AutoSpill is available through the R package autospill, available at the github repository https://github.com/carlosproca/autospill

For manuscripts utilizing custom algorithms or software that are central to the research but not yet described in published literature, software must be made available to editors and reviewers. We strongly encourage code deposition in a community repository (e.g. GitHub). See the Nature Research guidelines for submitting code & software for further information.

Data

Policy information about availability of data

All manuscripts must include a data availability statement. This statement should provide the following information, where applicable:

- Accession codes, unique identifiers, or web links for publicly available datasets
- A list of figures that have associated raw data
- A description of any restrictions on data availability

The raw data for the eight analyzed datasets is available at FlowRepository (https://flowrepository.org), with ids FR-FCM-Z2SV (Be1), FR-FCM-Z2ST (HS1 & HS2), FR-FCM-Z2SS (MM1), FR-FCM-Z2SW (MM2), FFR-FCM-Z2SJ (MM3), FRFCM-Z2SK (MM4), and FR-FCM-Z2SL (MM5). Note that the compensation controls for the MM2 dataset are the MM1 dataset.

Field-specific reporting

Please select the one below that is the best fit for your research. If you are not sure, read the appropriate sections before making your selection.

- [x] Life sciences
- [ ] Behavioural & social sciences
- [ ] Ecological, evolutionary & environmental sciences

For a reference copy of the document with all sections, see nature.com/documents/nr-reporting-summary-flat.pdf

Life sciences study design

All studies must disclose on these points even when the disclosure is negative.

Sample size	Data used in the manuscript was repurposed from prior experiments. >1000 test datasets were analysed to ensure that the method worked on typical datasets. 35 datasets are included on the website: https://autospill.vib.be/public/#/examples to cover multiple examples created on multiple machines. 7 datasets are shown in more detail, as representative of the different protocols used for mouse and human cells in the test datasets.
Data exclusions	No data was excluded
Replication	NA: Manuscript is bioinformatic in nature, primary data was not generated for the manuscript
Randomization	NA: Manuscript is bioinformatic in nature, primary data was not generated for the manuscript
Blinding	Investigators were blinded to sample grouping during data collection and processing.

Reporting for specific materials, systems and methods

We require information from authors about some types of materials, experimental systems and methods used in many studies. Here, indicate whether each material, system or method listed is relevant to your study. If you are not sure if a list item applies to your research, read the appropriate section before selecting a response.

Materials & experimental systems

n/a	Involved in the study	
[ ]	[x]	Antibodies
[x]	[ ]	Eukaryotic cell lines
[x]	[ ]	Palaeontology and archaeology
[x]	[ ]	Animals and other organisms
[x]	[ ]	Human research participants
[x]	[ ]	Clinical data
[x]	[ ]	Dual use research of concern

Methods

n/a	Involved in the study	
[x]	[ ]	ChIP-seq
[ ]	[x]	Flow cytometry
[x]	[ ]	MRI-based neuroimaging

Antibodies

Antibodies used	Complete list of all antibodies used is contained in the methods section.
Validation	Antibodies used as commercially validated.

Flow Cytometry

Plots

Confirm that:

- [x] The axis labels state the marker and fluorochrome used (e.g. CD4-FITC).
- [x] The axis scales are clearly visible. Include numbers along axes only for bottom left plot of group (a 'group' is an analysis of identical markers).
- [x] All plots are contour plots with outliers or pseudocolor plots.
- [x] A numerical value for number of cells or percentage (with statistics) is provided.

Methodology

Sample preparation	Peripheral blood mononuclear cells (PBMC) were isolated from heparinized blood samples of human healthy donors using Ficoll-Paque density centrifugation (MP biomedicals), frozen and then stored in liquid nitrogen. Frozen PBMCs were thawed

and counted, and cell concentration was adjusted to 1x10^6 for each single-color control. Cells were plated in a V-bottom 96-well plate, washed once with PBS (Fisher Scientific) and stained with live/dead marker and fluorochrome-conjugated antibodies against surface markers. Samples were stained for 60 min at 4C, washed twice in PBS/1% FBS (Tico Europe), and then fixed and permeabilized with Foxp3 Transcription Factor Staining Buffer Set (eBioscience), according to manufacturer's instructions. Cells were stored overnight at 4C and were then acquired on a Symphony flow cytometer with Diva software (BD Biosciences). A minimum of 5 x 10^4 events were acquired for each sample.Splenocytes from C57Bl/6 mice were disrupted with glass slides, filtered through 100 um mesh, and red blood cells lysed. Cells were fixed and permeabilized with Foxp3 Transcription Factor Staining Buffer Set (eBioscience) according to the manufacturer's instructions, and stained overnight at 4C. Samples were acquired on a Symphony flow cytometer (BD Biosciences).

Instrument

Symphony flow cytometer or Yeti/ZE5 flow cytometer, as described per dataset in the methods

Software

Source code for AutoSpill is available through the R package autospill, available at the github repository https://github.com/carlosproca/autospill, which includes batch code that reproduces the reported results for the datasets MM1, HS1, HS2, and Be1. The R package is also available in the Supplementary Information as Supplementary Data.

Cell population abundance

NA: samples were not sorted

Gating strategy

The initial gate was calculated independently for each control, over the 2d-density of events on forward and side scatter (FSC-A and SSC-A parameters). To robustly detect the population of interest, two tessellations were successively carried out to isolate the desired density peak. First, data was trimmed on extreme values (1% and 99%). Then, maxima were located numerically by a moving average (window size 3) on a soft estimation of the 2d-density (bandwidth factor 3). Maxima were used to generate non-overlapping tiles covering the entire 2d dataset (tessellation). The first tessellation was carried out on these density maxima, and the tile corresponding to the highest maximum was selected, ignoring peaks with lower values of both FSC-A and SSC-A (less than 5% of range). A rectangular region in the FSC-A/SSC-A-plane was chosen by using the median and the mean absolute deviation of the events contained in the selected tile. A second, finer 2d-density estimation (bandwidth factor 2) was obtained on this region, followed again by numerical detection of maxima (window size 2) and tessellation by the maxima. A final 2d-density estimation (bandwidth factor 1) was obtained on the tile containing the highest maximum, with the gate being defined as the convex hull enclosing the points that belonged to this tile and had a density larger than a threshold (33% of range).

☒ Tick this box to confirm that a figure exemplifying the gating strategy is provided in the Supplementary Information.
